# *Ginseng* and *Polygonum multiflorum* formula protects brain function in Alzheimer’s disease

**DOI:** 10.3389/fphar.2025.1461177

**Published:** 2025-02-20

**Authors:** Jing-Jing Liu, Feng Wei, Ya-Dan Wang, Jing Liu, Bei-Lei Xu, Shuang-Cheng Ma, Jian-Bo Yang

**Affiliations:** ^1^ National Institutes for Food and Drug Control, Beijing, China; ^2^ School of Pharmacy, Harbin University of Commerce, Harbin, China; ^3^ Kangtai Medical Testing Services Hebei Co., Ltd., Langfang, Hebei, China; ^4^ Chinese Pharmacopoeia Commission, Beijing, China

**Keywords:** ginseng and *Polygonum multiflorum*, Alzheimer’s disease, senescence, Sirt1, gut microbiota

## Abstract

**Background:**

Alzheimer’s disease (AD) is a progressive neurodegenerative disorder with no effective treatment currently available. The *Panax ginseng* C.A.Mey. and *Polygonum multiflorum* Thunb. formula (GSPM) has shown potential neuroprotective effects, but its therapeutic efficacy and underlying mechanisms in AD remain unclear and require further investigation.

**Methods:**

In this study, senescence-accelerated mouse prone 8 (SAMP8) mice, an AD model, were treated with GSPM (low: 117 mg/kg, high: 234 mg/kg) or donepezil (1.3 mg/kg) via gavage for 2 months. Cognitive function was assessed using the Morris water maze. Hippocampal morphology was evaluated by H&E staining, and neuronal apoptosis was detected by TUNEL assay. Microgliosis and astrogliosis were analyzed by Iba1 and GFAP immunohistochemistry. Levels of phosphorylated Tau, Aβ1-42, Aβ1-40, inflammatory cytokines, oxidative stress markers, and senescence markers were measured. Gut microbiota composition was analyzed by 16S rRNA sequencing. *In vitro*, the effects of GSPM were evaluated in Aβ1-42-stimulated HT22 hippocampal neurons. Cell viability was assessed via CCK-8, and apoptosis was detected by flow cytometry. The AMPK/Sirt1 pathway was investigated by Western blotting, and SIRT1-dependent effects were evaluated following EX527 treatment, a SIRT1 inhibitor.

**Results:**

GSPM treatment improved cognitive function, reduced hippocampal tissue damage, and decreased neuronal apoptosis in AD mice. It alleviated neuroinflammation by reducing microgliosis and astrogliosis and lowered the levels of p-Tau protein and Aβ accumulation in both the hippocampus and cerebrospinal fluid. Additionally, GSPM reversed the enhanced inflammation, oxidative stress, and neuronal senescence observed in AD mice. Furthermore, GSPM modulated gut microbiota composition by reducing microbial diversity and restoring the *Firmicutes*/*Bacteroidetes* ratio to levels similar to those in control mice. GSPM increased the abundance of *Lactobacillus*, which was negatively correlated with inflammation, Aβ1-42, p-Tau, and senescence markers. It also decreased the abundance of bacteria, such as *Oscillibacter*, *Helicobacter*, and *Odoribacter*, which are associated with inflammation, oxidative stress, and neuronal senescence. In line with *in vivo* findings, GSPM increased cell viability, reduced apoptosis, and alleviated oxidative stress in Aβ1-42-stimulated HT22 hippocampal neurons. It also decreased the production of pro-inflammatory cytokines and reduced expression of senescence markers *in vitro*. Furthermore, GSPM restored AMPK phosphorylation and Sirt1 expression in neurons. Notably, inhibition of Sirt1 by EX527 reversed the neuroprotective effects of GSPM.

**Conclusion:**

Our data demonstrated that GSPM exhibits protective effects on AD via suppressing the inflammation, oxidation, and senescence, possibly through regulating the Sirt1 signaling. These findings provided a novel therapeutic approach for AD.

## Introduction

Alzheimer’s disease (AD) is an age-related neurodegenerative disorder for which no effective treatments are currently available (Scheltens et al., 2021). The disease is characterized by the deposition of amyloid-β (Aβ) plaques, the formation of neurofibrillary tangles, and the loss of neurons and synapses (Hampel et al., 2021). These pathological changes gradually cause a decline in cognitive function, memory, and daily living abilities, often accompanied by local inflammation, activation of microglia and astrocytes, neuritic degeneration, and lysosomal dysfunction (Rostagno, 2022; Sun et al., 2018). Additionally, AD is frequently associated with the accumulation of senescent cells, which exhibit phenotypic characteristics such as pro-inflammatory cytokine secretion, oxidative stress, and resistance to apoptosis (Zhu et al., 2024). Current treatments for AD primarily focus on alleviating symptoms, such as improving memory and cognitive function; however, no curative treatment is available. Most clinical drugs, including the FDA-approved Aducanumab and Lecanemab, provide limited symptomatic relief, but they are often accompanied by significant side effects, such as headaches and nausea (Gorthi and Gupta, 2023). These side effects can negatively impact patients’ quality of life and may even lead to treatment discontinuation. Therefore, there is an urgent need for the development of novel therapeutic agents that not only effectively alleviate symptoms but are also suitable for long-term use. Given the multifactorial nature of AD, a single-target therapeutic approach may be insufficient to achieve optimal therapeutic outcomes. Traditional Chinese medicine (TCM), with its ability to target multiple pathways and mechanisms, offers promising potential for AD treatment.

TCM and its active extracts have been shown to enhance cognitive function and offer neuroprotective effects (Jarrell et al., 2018; Sreenivasmurthy et al., 2017). TCM offers multiple benefits for the treatment of AD through mechanisms such as modulating neurotransmitter activity, reducing Aβ deposition, inhibiting acetylcholinesterase activity, preventing neuronal apoptosis, and enhancing antioxidant activity (Jarrell et al., 2018; Sreenivasmurthy et al., 2017). For example, botanical drugs such as *Polygala tenuifolia* Willd. [Polygalaceae; Polygalae tenuifoliae radix], *Morinda officinalis* F.C.How [Rubiaceae; Morindae officinalis radix], *Panax ginseng* C.A.Mey. [Araliaceae; Panacis ginseng radix], *Salvia miltiorrhiza* Bunge [Lamiaceae; Salviae miltiorrhizae radix et rhizoma], and their active extracts have demonstrated positive effects in the prevention and treatment of AD, primarily through mechanisms like increasing acetylcholine levels, reducing Aβ deposition, inhibiting neuroinflammation, and decreasing tau protein expression (Chong et al., 2019; Razgonova et al., 2019; Deng et al., 2020).

*Panax ginseng* C.A.Mey. [Araliaceae; Panacis ginseng radix] and *Polygonum multiflorum* Thunb. [Polygonaceae; Polygoni multiflori radix] are commonly used botanical drugs in TCM, known for their significant neuroprotective and cognitive-enhancing effects. *Panax ginseng* modulates the central nervous system, improves cerebral blood flow, and enhances antioxidant function, making it widely used in the prevention and treatment of AD (Yu, 2005). *Polygonum multiflorum*, renowned for its anti-aging, liver and kidney tonifying, and nerve regeneration properties, also plays an important role in AD treatment (Chi et al., 2022). Their combination, in the form of *ginseng* and *P. multiflorum* formula (GSPM), may exert a synergistic effect through multiple mechanisms, such as improving circulation, regulating neuroinflammation, and enhancing antioxidant defenses, potentially delaying neurodegenerative changes and promoting brain health (Chi et al., 2022; Liu et al., 2024). However, the precise mechanisms of action remain to be further investigated.

Silent Information Regulator 1 (Sirt1), a NAD + -dependent deacetylase, is a pivotal regulator of various biological processes, including cellular stress resistance, DNA repair, inflammation, and metabolism (Gomes et al., 2018). Recent studies have highlighted Sirt1’s neuroprotective effects, suggesting that it may serve as a novel therapeutic target for AD. Sirt1 regulates several signaling pathways that influence neuroinflammation, oxidative stress, and neurodegeneration, all of which are key features of AD pathology (Wong and Tang, 2016; Wang R. et al., 2022). Natural molecules, such as icariin, resveratrol, quercetin, salidroside, patchouli, ligustilide, schisandrin, curcumin, and betaine, have been shown to regulate Sirt1 and its downstream pathways, offering potential therapeutic benefits in AD treatment (Zhang and Tang, 2023). It remains unknown whether GSPM can exert its anti-Alzheimer effects by regulating the SIRT1 signaling pathway.

In this study, we analyzed the protective effects of GSPM in AD models using *in vivo* and *in vitro* approaches. We examined the inflammatory and oxidative condition, and neuron senescence, and explored the molecular mechanisms related to Sirt1.

## Materials and methods

### Preparation of GSPM

Panax ginseng C.A.Mey. [Araliaceae; Panacis ginseng radix] and Polygonum multiflorum Thunb. [Polygonaceae; Polygoni multiflori radix] were purchased from Tongrentang Pharmacy in Beijing, and authenticated by Professor Kang-shuai from the National Institutes for Food and Drug Control. The *ginseng* and *P. multiflorum* were powdered and sieved, then weighed in a 2:3 ratio. 300 g of the mixed medicinal powder was weighed, and 3 L of 70% ethanol (analytical grade, National Pharmaceutical Group Chemical Reagent Co., Ltd.) was added. The mixture was soaked overnight at room temperature, followed by 24 h of percolation. The permeate filtrates were combined and concentrated into a paste with a relative density of 1.25–1.30. The paste was then dried under reduced pressure at 60°C to form a dry extract, which was ground into a fine powder to obtain GSPM. Before HPLC analysis, the GSPM formulation was diluted to a concentration of 10 mg/mL (w/v, crude drug/water) and filtered using a 0.22 μm membrane filter.

### Mouse model

All animal experiments were conducted following the guidelines set by the Ethic Committee of Kangtai Medical Testing Services Hebei Co., Ltd. Six-month-old male senescence-accelerated mouse prone 8 (SAMP8) and senescence-accelerated mouse resistant 1 (SAMR1) mouse were procured from Peking University Health Science Center (Certificate No. SCXK(Jing)2021-0013, Beijing, China). SAMR1 mice, serving as the aging-resistant control, were given sodium carboxymethyl cellulose. The SAMP8 model group was randomly subdivided into the following groups: AD model group (sodium carboxymethyl cellulose), positive control (1.3 mg/kg donepezil daily), and GSPM treatment groups (low dose: 117 mg/kg, high dose: 234 mg/kg). Treatments lasted for 2 months. Following treatment, mice were anesthetized with isoflurane and transcardially perfused with PBS followed by 4% paraformaldehyde (PFA). Hippocampal tissues were cryoprotected in 30% sucrose, then sectioned horizontally into 15-μm thick slices using a microtome.

### Behavioral analysis

#### Morris water maze test

The Morris water maze test was performed to assess the learning and memory abilities of mice. In brief, a circular water pool was divided into four equal areas, with a platform placed in one of the areas. Swimming training was conducted four times daily for 7 days, with a maximum duration of 120 s to find the platform. During the training, the mice were placed at a random location and allowed to swim in search of the hidden platform. 24 h after the final trial, the escape latency (time taken to reach the platform) and the frequency of platform crossings were recorded daily for 5 days using a tracking system.

#### Histological analysis

Brain tissue slices were stained with hematoxylin and eosin (HE) to evaluate tissue damage. For immunofluorescence (IF) staining, tissues were incubated with 0.3% H_2_O_2_ for 10 min to block endogenous peroxidase activity, then probed overnight at 4°C with primary anti-Iba1 (1:200, Abcam, United States) and anti-GFAP antibody (1:200, Abcam, United States). After washing, tissues were incubated with Alexa Fluor 488-conjugated or Alexa Fluor 633-conjugated secondary antibody at room temperature for 1 hour. The nucleus was labeled with DAPI (Beyotime, China). To detect cell apoptosis, TdT-mediated dUTP-biotin nick end-labeling (TUNEL) assay was performed according to the manufacturer’s instructions using a TUNEL staining reagent (Beyotime, China). Fluorescence images were captured using confocal microscopy (Carl Zeiss, Germany).

#### Cell line and treatment

Mouse hippocampal neuron HT22 cells were obtained from National Infrastructure of Cell Line Resource (Beijing, China) and cultured in Dulbecco’s modified Eagle’s medium (DMEM) with 10% fetal bovine serum (FBS, Hyclone, United States), 100 μg/mL streptomycin, and 100 U/mL penicillin in a humidified 37°C incubator with 5% CO_2_. Aβ1-42 was added to the culture medium at a final concentration of 20 µM and incubated for 48 h for subsequent *in vitro* experiments. When required, Sirt1 inhibitor EX527 (5 µM) was used to treat HT22 cells for 8 h.

#### Detection of oxidative stress

The hippocampus tissue and HT22 cells were homogenized in PBS and centrifuged at 12,000 g for 10 min at 4°C. The supernatant was collected for the measurement of malondialdehyde (MDA), superoxide dismutase (SOD), glutathione (GSH), and Total antioxidant capacity (T-AOC) using commercial detection kits (Nanjing Jiancheng, China).

#### Enzyme-linked immunosorbent assay (ELISA)

Hippocampus tissues were washed with PBS, homogenized in lysis buffer, sonicated, and then centrifuged at 100,000 rpm for 60 min. The supernatant was collected for analysis. The levels of pro-inflammatory cytokines including tumor necrosis factor-α (TNF-α), interleukin (IL)-1β, IL-6, and 42 amino acid end of Aβ (Aβ42) in both hippocampus tissues and HT22 cell culture medium were measured using commercial ELISA kits (Elabscience, United States) according to manufacturer’s protocols.

#### Quantitative real-time PCR (qRT-PCR)

Brain tissues were homogenized in liquid nitrogen, and total RNA was extracted by adding 1 mL of Trizol reagent (Thermo, United States). A total of 1 µg RNA was used to synthesize cDNA using a PrimeScript™ RT Master Mix (Takara, Kyoto, Japan). The cDNA was then amplified using the One Step SYBR Ex Taq™ qRT-PCR kit (Takara) according to the manufacturer’s instructions. Gene expression levels were quantified using the 2^−ΔΔCt^ method and normalized to β-actin level.

#### Western blotting assay

Total proteins were extracted from brain tissues and HT22 cells with RIPA lysis buffer (Beyotime, China) and centrifuged at 12,000 g for 20 min. Protein concentration was determined using a BCA kit (Beyotime). A total of 50 µg proteins was loaded onto a 10%∼12% SDS-PAGE gel, then transferred to PVDF membranes. The membranes were blocked with 5% skimmed milk at room temperature for 1 h, followed by overnight incubation at 4°C with primary antibodies against AMPK, p-AMPK, Sirt1, TNF-α, IL-1β, IL-6, P21, P16, P53, and β-actin. Subsequently, the membranes were incubated with HRP-conjugated secondary antibodies (anti-mouse or anti-rabbit) at room temperature for 1 h and visualized using an ECL reagent (Millipore, United States). Protein bands were captured with an imaging system (Tanon, China).

#### Cell counting kit 8 (CCK-8) experiment

HT22 cells were seeded into 96-well plates at a density of 10,000 cells per well and placed at 37°C incubator for 12 h. Therapeutic reagents, including GSPM (final concentration 10 mg/mL) and EX527 (final concentration 5 µM), were added to the culture medium and incubated for 24 h. Following treatment, CCK-8 reagent was added to each well and incubated for an additional 2 h at 37°C. Absorbance values at 450 nm were measured, and cell viability was calculated.

#### Cell cycle analysis

Cell cycle analysis was performed using a cell cycle detection kit (Beyotime) according to the manufacturer’s instructions. Briefly, cells were collected, washed with PBS, and fixed with ice-cold 70% ethanol at 4°C for 12 h. After fixation, cells were centrifuged, resuspended in PBS containing PI reagent, and incubated at 37°C for 30 min. Cells were then washed and resuspended in PBS. Cell cycle distribution was measured by flow cytometer (BD Bioscience, United States) and analyzed with Flowjo software.

#### SA-β-gal staining

Cell senescence was assessed using an SA-β-gal staining kit (Beyotime) according to the manufacturer’s protocol. Briefly, cells were seeded into the 6-well plate, fixed with fixation solution for 15 min, and then stained with SA-β-gal solution at 37°C overnight. Images were captured using an optical microscope (Leica, Germany).

#### High-performance liquid chromatography (HPLC) analysis

High-performance liquid chromatography (HPLC) was carried out with a Waters Acquity HPLC system equipped with XBridge@ Shield BEH 18 chromatographic column (3.0 mm × 150 mm, 2.5 μm). The mobile phase consisted of acetonitrile (A) and 0.1% phosphoric acid solution (B). The gradient elution conditions were as follows: 0–3 min, 2% A; 3–6 min, 2%–5% A; 6–15 min, 5%–15% A; 15–26 min, 15%–30% A; 26–28.5 min, 35%–40% A; 28.5–30 min, 35%–40% A; 30–31 min, 40%–42.5% A; 31–34 min, 42.5%–55.5% A; 34–40.5 min, 55.5%–74% A; 40.5–45 min, 74%–95% A. The flow rate was set at 1 mL/min, with an injection volume of 5 μL, and the column temperature was maintained at 35°C. Chromatographic peaks were detected at wavelengths of 202 nm and 270 nm.

#### Fecal microbiome preprocessing and data analysis

At the end of the experiment, pellet samples were collected and bacterial genomic DNA was extracted from each fecal sample using the PF Mag-Bind Stool DNA Kit (Omega Bio-tek, Georgia, United States). Bioinformatic analysis of the gut microbiota was carried out using the Majorbio Cloud platform (https://cloud.majorbio.com). Based on the OTUs information, rarefaction curves and alpha diversity indices, including the Shannon and Sobs indices, were calculated using Mothur v1.30.2 (Schloss et al., 2009). The similarity among microbial communities in different samples was assessed by principal coordinate analysis (PCoA) based on Bray-Curtis dissimilarity, using the Vegan v2.4.3 package. The PERMANOVA test was conducted to assess the proportion of variation explained by the treatment and its statistical significance using the Vegan v2.4.3 package. Correlations between two nodes were considered statistically significant and robust if the Spearman’s correlation coefficient was greater than 0.6 or less than −0.6, and a P-value less than 0.05.

#### Statistics

Data are presented as means ± SD of three independent tests and were analyzed using the SPSS 21.0 software (La Jolla, CA, United States). Comparisons among two or more groups were conducted with Student's *t*-test or one-way analysis of variance (ANOVA) followed by Tukey’s test. *P* < 0.05 is set as statistically significant.

## Results

### HPLC analysis of GSPM

The metabolites of GSPM are chemically characterized by the HPLC method. Chromatograms of the *ginseng* and *P. multiflorum* extract were recorded at 270 nm and 202 nm, and compared with the mixed reference standard chromatograms. Through comparison, 13 metabolites were identified, including 7 metabolites from *P. multiflorum* and 6 metabolites from *ginseng*. At 270 nm, the common peaks include: cis-2,3,5,4′-tetrahydroxystilbene-2-O-β-D-glucoside (peak 1), polydatin (peak 2), trans-2,3,5,4′-tetrahydroxystilbene-2-O-β-D-glucoside (peak 3), emodin-8-O-β-D-glucoside (peak 6), physcion-8-O-β-D-glucoside (peak 7), emodin (peak 12), and physcion (peak 13). At 202 nm, the common peaks include: ginsenoside Rg1 (peak 4), ginsenoside Re (peak 5), 20(S)-Ginsenoside Rg2 (peak 8), ginsenoside Rb1 (peak 9), ginsenoside Ro (peak 10), and ginsenoside Rd (peak 11) (Supplementary Figure S1).

### GSPM improves neuron damage in AD mouse model

**FIGURE 1:**
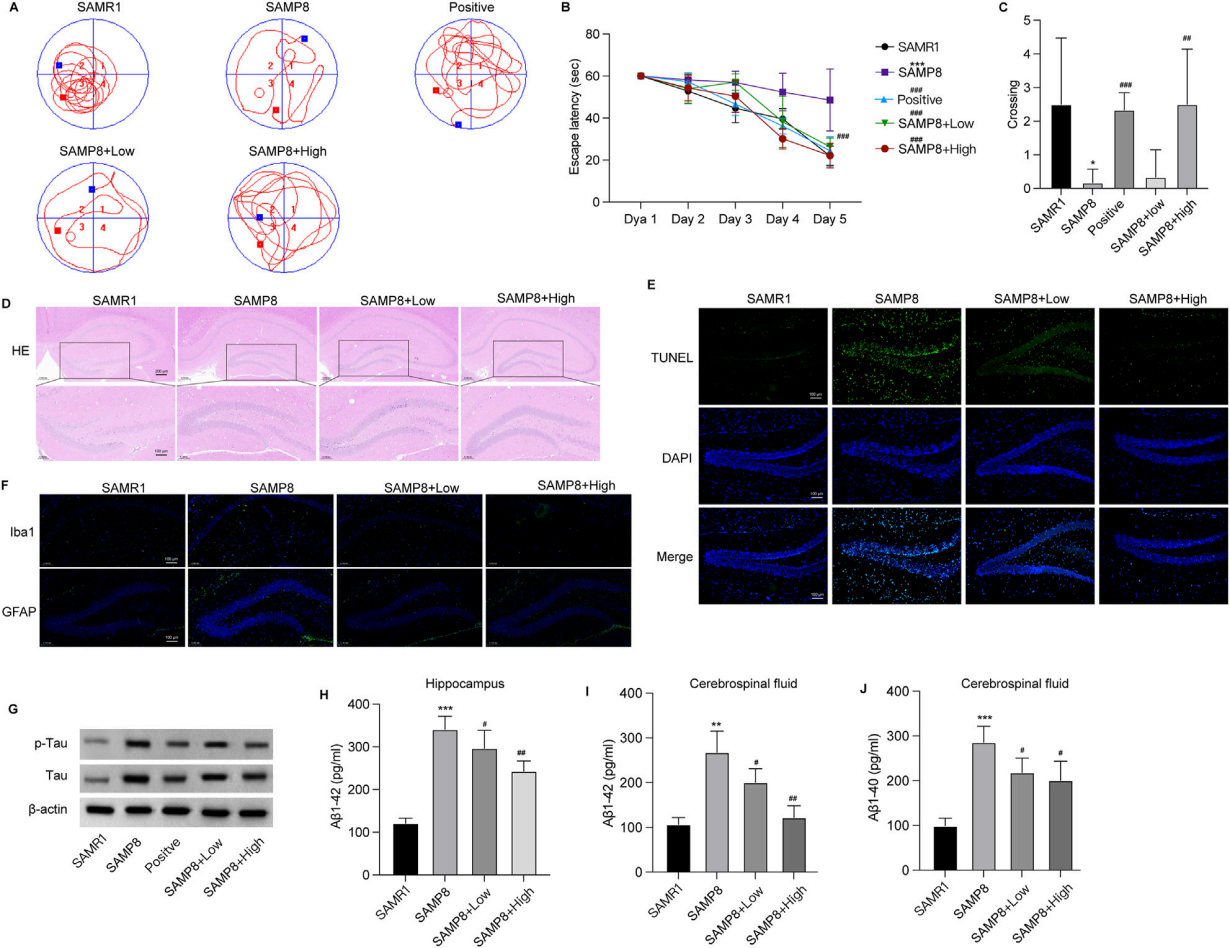
GSPM alleviates neuron damage in the AD mouse model. **(A)** Swimming trajectory of mice in Morris water maze test. **(B)** Escape latency of mice over 5 consecutive days **(C)** Frequency of crossing the platform of mice in Morris water maze test. **(D)** HE staining of hippocampus tissues. **(E)** TUNEL staining to assess apoptotic neurons in the hippocampus. **(F)** Representative Immunofluorescence staining of astrocytes and microglia utilizing anti‐GFAP and anti‐Iba1 antibodies in mouse hippocampal tissue. **(G)** Western blot analysis of total and p-Tau expression. **(H)** The accumulation of Aβ1-42 in hippocampus. **(I–J)** Aβ1-42 and Aβ1-40 levels in cerebrospinal fluid measured by ELISA. *p < 0.05, **p < 0.01, ***p < 0.001 vs. SAMR1 mice; #p < 0.05, ##p < 0.01, ###p < 0.001 vs. SAMP8 mice.

An AD mouse model was established to evaluate the *in vivo* effects of GSPM. In the Morris water maze test, the model group exhibited learning and memory deficits compared to the SAMR1 group, while high-dose GSPM treatment alleviated cognitive impairment, as evidenced by the decreased escape latency to find the platform (Figures 1A, B) and increased platform crossing number on day 5 after platform removal (Figure 1C). HE staining demonstrated that high dose GSPM treatment could notably suppress hippocampus tissue damage of AD mice (Figure 1D). The TUNEL assay revealed significant neuronal death in the hippocampus of AD mice, which was significantly reduced by low-dose GSPM treatment, with further suppression at higher doses (Figure 1E). Moreover, GSPM treatment, both at low and high doses, significantly reduced the proportion of AD plaque-associated microglia (Iba1 expression) and astrocytes (GFAP activation) (Figure 1F), indicating alleviated neurological inflammation. Furthermore, GSPM treatment, at both low and high doses, significantly reduced the elevated levels of total and phosphorylated Tau protein in AD mice (Figure 1G). Consistently, GSPM treatment dose-dependently reduced Aβ1-42 accumulation in the hippocampus (Figure 1H) and Aβ1-42 and Aβ1-40 levels (Figures 1I, J) in cerebrospinal fluid of AD mice. These data indicated that GSPM could alleviate brain tissue damage and inflammation in AD mice.

### GSPM alleviates inflammation, oxidative stress and neuron senescence in AD

**FIGURE 2:**
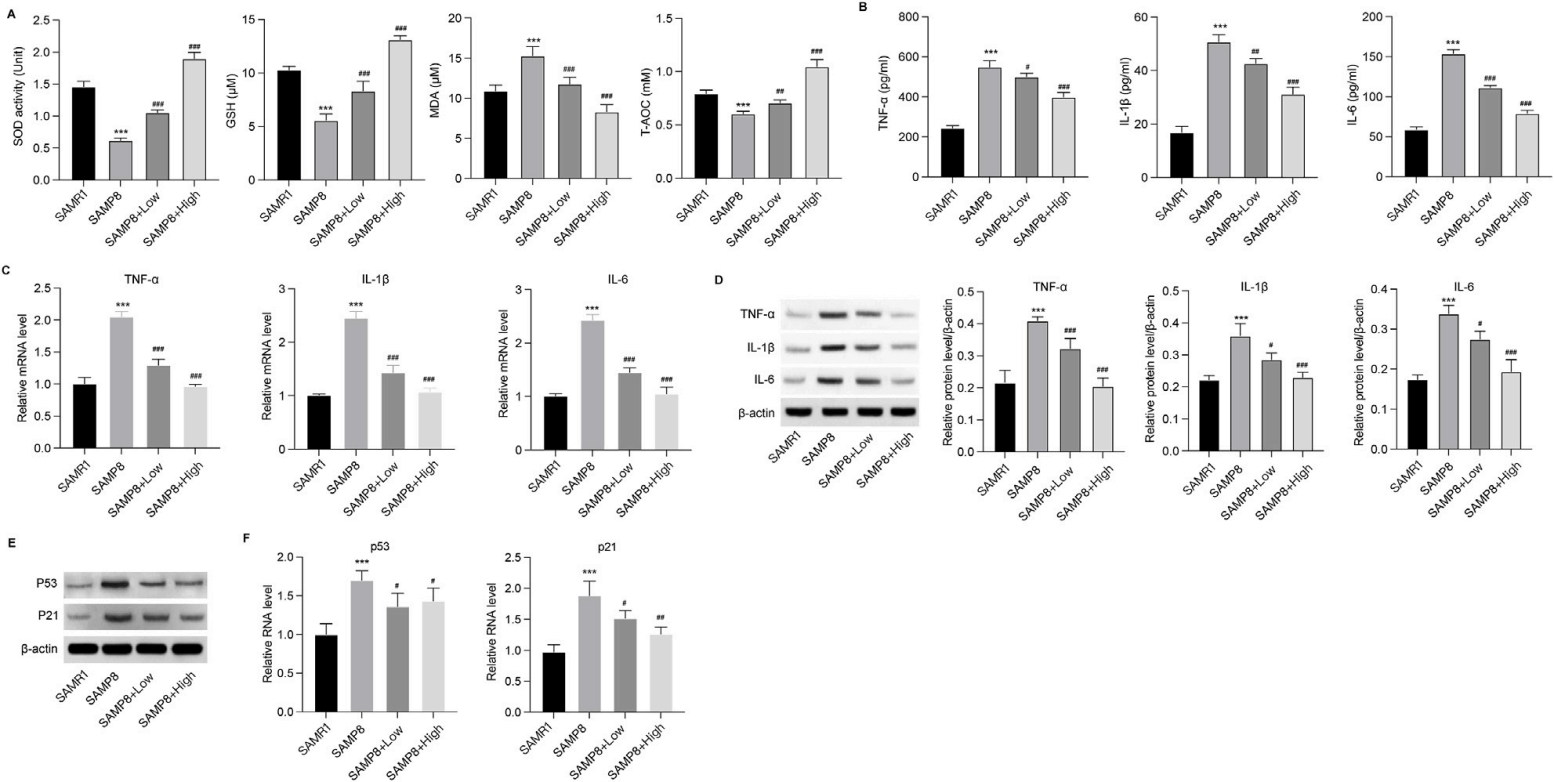
GSPM alleviates inflammation and oxidative stress in AD. **(A)** Secretion of SOD, GSH, MDA, and T-AOC in hippocampus tissues was evaluated using commercial assay kits. **(B)** Accumulation of inflammatory cytokines, including TNF-α, IL-1β, and IL-6, in hippocampus tissues was measured by ELISA. **(C)** mRNA expression levels of TNF-α, IL-1β, and IL-6 in hippocampus of AD mice were detected by qRT-PCR. **(D)** Protein expression levels of TNF-α, IL-1β, and IL-6 in hippocampus were measured by Western blotting. **(E)** Protein and **(F)** mRNA expression levels of P53 and P21 in hippocampus of mice determined by Western blotting and. qRT-PCR, respectively *p < 0.05, **p < 0.01, ***p < 0.001 vs. SAMR1 mice; #p < 0.05, ##p < 0.01, ###p < 0.001 vs. SAMP8 mice.

The effects of GSPM on brain tissue inflammation and oxidative stress were subsequently evaluated in AD mice. In the hippocampus, levels of antioxidant agents, including SOD, GSH, and T-AOC, were significantly reduced, while the oxidation metabolite MDA was elevated in AD mice. GSPM treatment reversed these changes in a dose-dependent manner (Figure 2A). Additionally, GSPM treatment dose-dependently inhibited the secretion of inflammatory cytokines TNF-α, IL-1β, and IL-6 in the hippocampus of AD mice (Figure 2B), and similarly suppressed their mRNA and protein expression levels (Figures 2C, D). Furthermore, the expression of cell senescence markers P21 and P53 was significantly induced in AD mice and was dose-dependently reduced by GSPM treatment (Figures 2E, F). These data indicated that GSPM could alleviate inflammation, oxidative stress, and cell senescence in AD mice.

### Microbiota analysis on GSPM-treated mice

**FIGURE 3:**
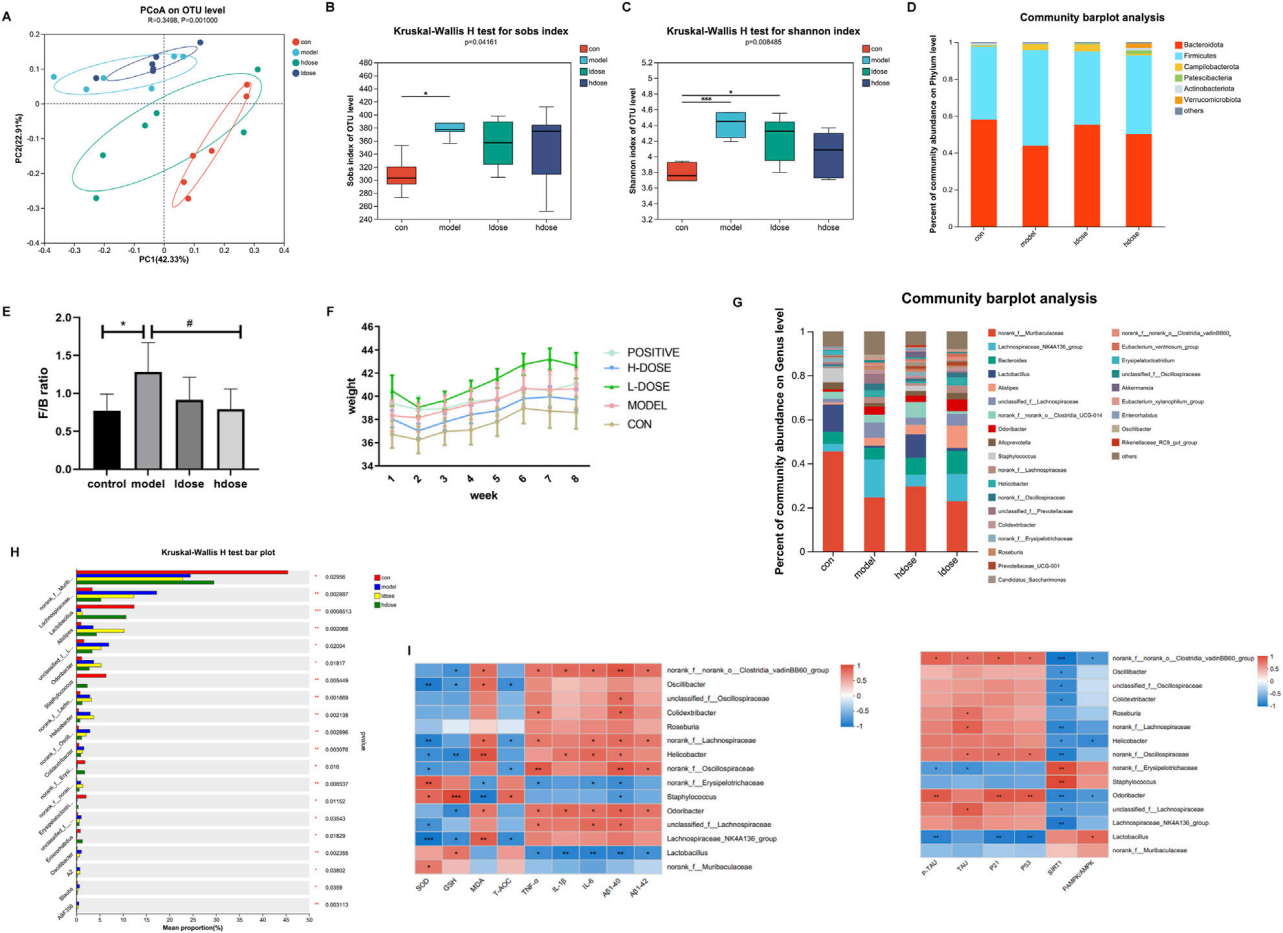
Microbiota and metabolomics analysis on GSPM-treated mice. **(A)** PCoA of microbiota among the five groups. **(B, C)** Comparison of alpha diversity of intestinal flora among the four groups based on the Sob and Shannon indices. **(D)** The fecal microbial distribution at phylum level. **(E)** The ratio of *Firmicutes* to *Bacteroidetes* (F/B) in different groups. **(F)** Mice body weight in different groups. **(G)** The fecal microbial distribution at genus level. **(H)** Top 15 bacterial colonies with p-value <0.05. **(I)** Heatmap visualizing the correlations between the gut microbiota and inflammatory factors (TNFα, IL-1β, IL-6), oxidative regulators (SOD, GSH, MDA, T-AOC), and senescence biomarkers (P21, P53). Con: SAMR1 mcie, Model: SAMP8 mice, hdose: high-dose of GSPM, ldose, low-dose of GSPM. *p < 0.05, **p < 0.01, ***p < 0.001 vs. cont group; #p < 0.05, ##p < 0.01, ###p < 0.001 vs. model group.

To assess the impact of GSPM on gut microbiota, 16S rRNA sequencing was conducted on fecal samples. PCoA analysis revealed that the bacterial diversity between aging and control mice was significantly separated, the high-dose GSPM group resembled the control group, and the low-dose GSPM group was closer to the model group (ANOSIM R statistic = 0.3498 p-value = 0.001) (Figure 3A). The alpha diversity of aging AD mice was significantly higher than that of control mice, as indicated by Sobs and Shannon indices (Kruskal-Wallis, p = 0.0416, 0.008485), and GSPM treatment dose-dependently reduced microbial diversity in AD mice (Figures 3B, C). These findings suggest that the gut microbiota composition of aging AD mice differs significantly from that of control mice, and GSPM treatment modulates this microbiota.

At the phylum level, fecal microbiota in all groups was primarily composed of *Firmicutes*, *Bacteroidetes*, and *Campilobacterota* (Figure 3D). Compared to the control group, the model group showed a significant increase in *Campilobacterota* and *Firmicutes*, whereas the high-dose GSPM group exhibited a reduction in both phyla compared to the model group (Figure 3D). An increased *Firmicutes/Bacteroidetes* (F/B) ratio is associated with weakened epithelial tight junctions, facilitating the transfer of proinflammatory cytokines produced by pathogenic bacteria to the brain via the bloodstream or vagus nerve. Inflammation, driven by leaky gut syndrome, can also exacerbate cognitive decline. Notably, the F/B ratio was significantly higher in aging AD mice than in control mice (p < 0.05), while GSPM treatment dose-dependently restored this ratio (p < 0.05, Figure 3E). Furthermore, aging AD mice had significantly higher body weight when compared to control mice, and high-dose GSPM suppressed this weight gain (Figure 3F).

Genus-level microbiota analysis identified the top 15 most significantly altered taxa (p < 0.05) (Figures 3G, H). Correlation heatmap analysis revealed significant associations between gut microbiota composition and levels of inflammatory factors (TNF-α, IL-1β and IL-6), oxidative regulators (SOD, GSH, MDA and T-AOC), and senescence biomarkers (P21 and P53) (r < |0.6|, Figure 3I). Specifically, GSPM treatment increased *Lactobacillus* abundance, which was negatively correlated with levels of inflammatory factors, MDA, Aβ1-42, p-Tau, and senescence biomarkers (P21 and P53) and positively correlated with p-AMPK signaling. Moreover, the abundances of *Oscillibacter*, *Roseburia*, *Colidextribacter*, *Helicobacter*, *Odoribacter*, and *Lachnospiraceae* were reduced upon GSPM treatment. Among them, *Oscillibacter* was negatively correlated with levels of SOD, GSH, T-AOC and SIRT1; *Colidextribacter* was positively correlated with levels of Aβ1-42 and TNF-α and negatively correlated with SIRT1; *Helicobacter* was positively correlated with levels of IL-1β, IL-6, Aβ1-40 and negatively correlated with SIRT1 and p-AMPK/AMPK; *Odoribacter* was positively correlated with inflammatory factors, oxidative regulators, senescence biomarkers and Aβ1-40, Aβ1-42, and p-Tau and negatively correlated with SIRT1 and p-AMPK/AMPK. These data indicated that gut microbiota is closely correlated with inflammation, oxidative response, and AMPK-SIRT1 signaling pathway.

### GSPM protects neurons from inflammation and oxidative stress-induced death *in vitro*

**FIGURE 4:**
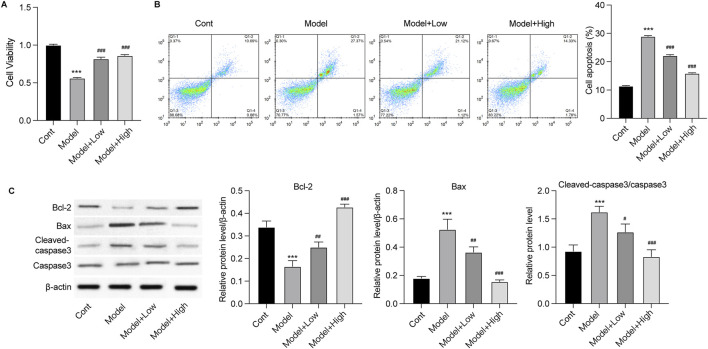
GSPM alleviates neuron death *in vitro*. HT22 cells were stimulated with Aβ1-42 and treated with GSPM at low and high doses. **(A)** Cell viability and **(B)** apoptosis were detected by CCK-8 and flow cytometry, respectively. **(C)** Protein levels of Bcl-2, Bax, cleaved-caspase 3, and caspase 3 were measured by Western blotting. *p < 0.05, **p < 0.01, ***p < 0.001 vs. SAMR1 group; #p < 0.05, ##p < 0.01, ###p < 0.001 vs. SAMP8 group.

**FIGURE 5:**
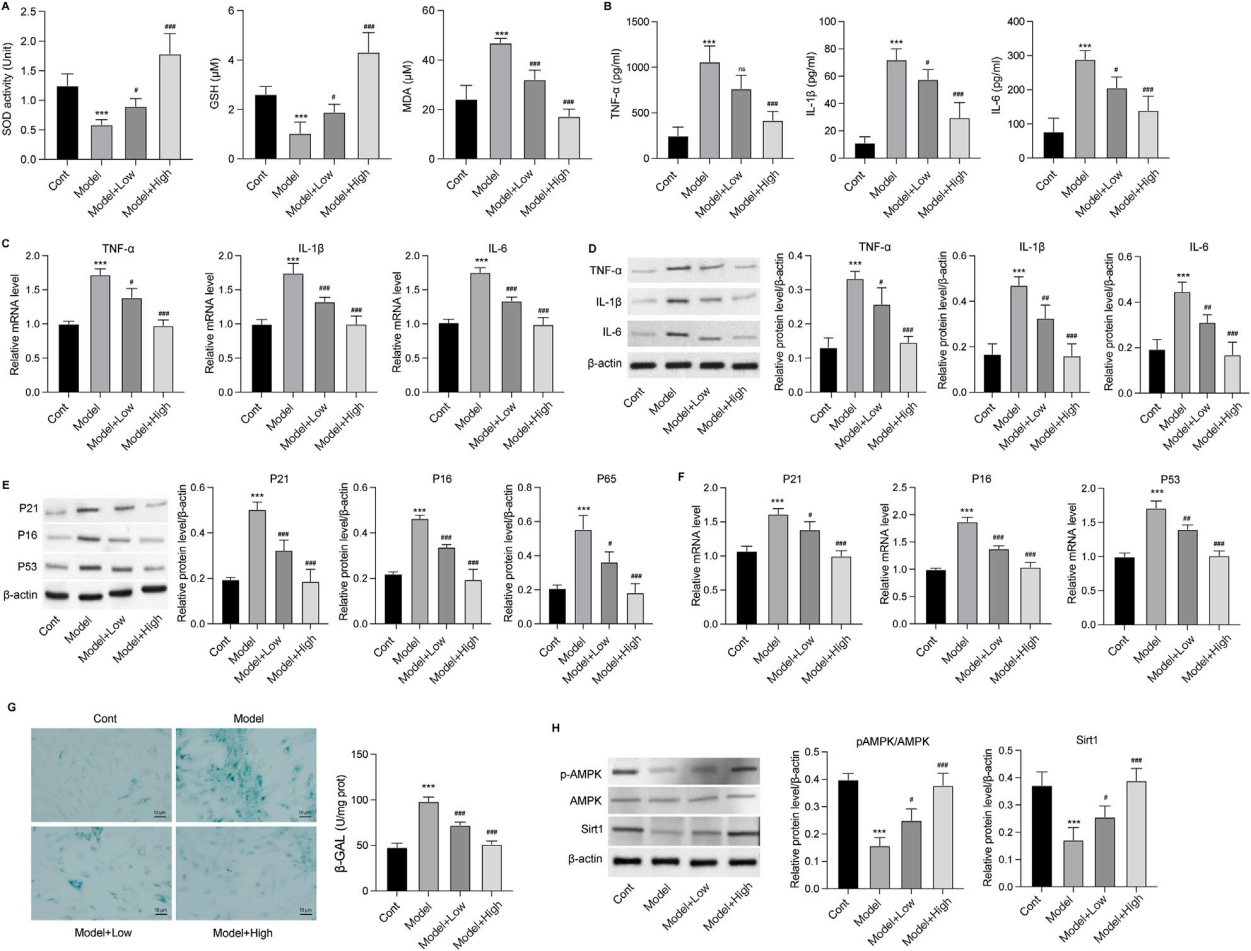
GSPM alleviates inflammation, oxidative stress, and senescence *in vitro*. HT22 cells were stimulated with Aβ1-42 and treated with GSPM at low and high doses. **(A)** The SOD activity, GSH and MDA level in HT22 neurons were evaluated using commercial assay kits. **(B)** Secretion levels, **(C)** mRNA expression levels, and **(D)** protein levels of TNF-α, IL-1β, and IL-6 were measured by ELISA, qPCR, and Western blotting, respectively. **(E)** Protein and **(F)** mRNA levels of P21, P16, and P53 were measured by Western blotting and qPCR assay, respectively. **(G)** The SA-β-gal activity of HT22 cells was detected via an SA-β-gal staining assay. **(H)** Protein levels of phosphorylated AMK, AMPK, and Sirt1 in HT22 cells were detected by Western blotting. *p < 0.05, **p < 0.01, ***p < 0.001 vs. SAMR1 group; #p < 0.05, ##p < 0.01, ###p < 0.001 vs. SAMP8 group.

To assess the protective effects of GSPM, we established an *in vitro* model using Aβ1-42-stimulated mouse hippocampal neurons HT22. As shown in Figure 4A, compared with the model group, GSPM treatment significantly increased the cell viability of HT22 cells in a dose-dependent manner. Additionally, GSPM treatment reduced cell apoptosis, as evidenced by increased Bcl-2 levels and decreased Bax and cleaved caspase-3 levels (Figures 4B, C). We next analyzed the expression of oxidative stress-related factors and inflammatory cytokines. As shown in Figure 5A, SOD activity and GSH levels were significantly reduced in the model group relative to the control group, while GSPM treatment dose-dependently restored these levels. Furthermore, GSPM treatment notably decreased the production, mRNA, and protein expression levels of pro-inflammatory cytokines TNF-α, IL-1β, and IL-6 when compared to the model group (Figures 5B–D).

### GSPM retards neuron senescence

**FIGURE 6:**
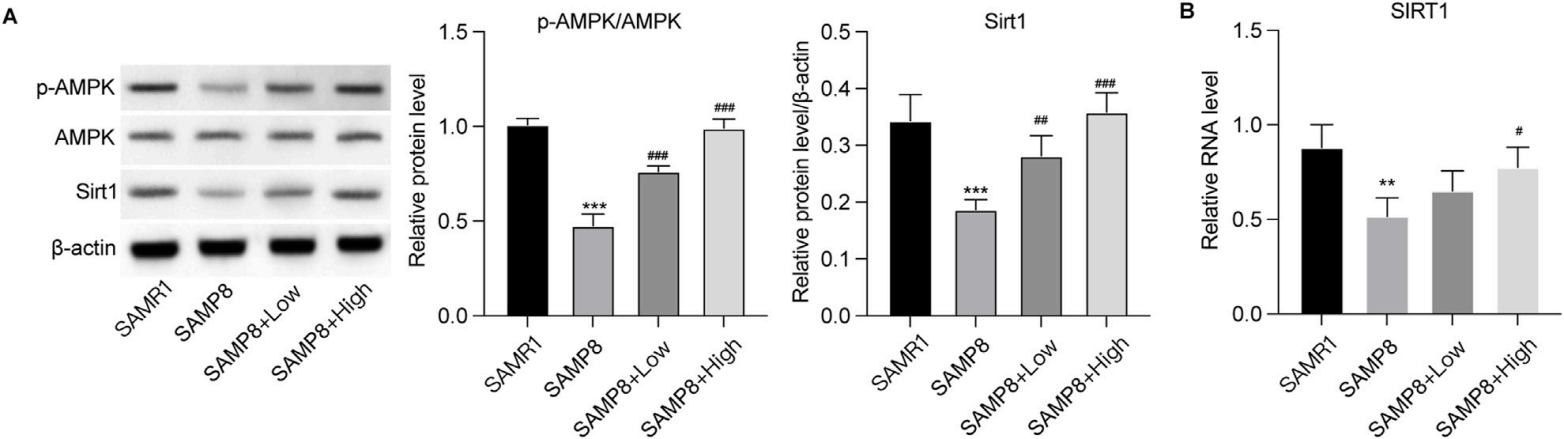
GSPM affects the AMPK/Sirt1 signaling *in vivo*. **(A)** Protein expression levels of phosphorylated AMK, AMPK, and Sirt1 in hippocampus tissues were detected by Western blotting. **(B)** mRNA level of SIRT in hippocampus tissues was measured by qPCR assay. *p < 0.05, **p < 0.01, ***p < 0.001 vs. SAMR1 group; #p < 0.05, ##p < 0.01, ###p < 0.001 vs. SAMP8 group.

The effects of GSPM on neuron senescence were subsequently evaluated. As shown in Figures 5E, F, the protein and mRNA expression levels of senescence markers (P21, P16, and P53) were significantly upregulated in the model group, while GSPM treatment could dose-dependently reduce their expression. SA-β-gal staining further demonstrated increased β-gal activity in Aβ1-42-stimulated HT22 cells, which was alleviated by GSPM (Figure 5G). Besides, the model group showed suppressed phosphorylation level of AMPK and reduced Sirt1 expression, whereas GSPM treatment restored both in HT22 cells (Figure 5H). Consistently, *in vivo* experiments demonstrated that the phosphorylation of AMPK and expression of Sirt1 were also decreased in the hippocampus of model mice compared to sham controls, and GSPM significantly elevated their levels (Figure 6). These data suggest that GSPM may protect neurons via regulating the Sirt1 signaling.

### GSPM protects neurons’ function via regulating Sirt1

**FIGURE 7:**
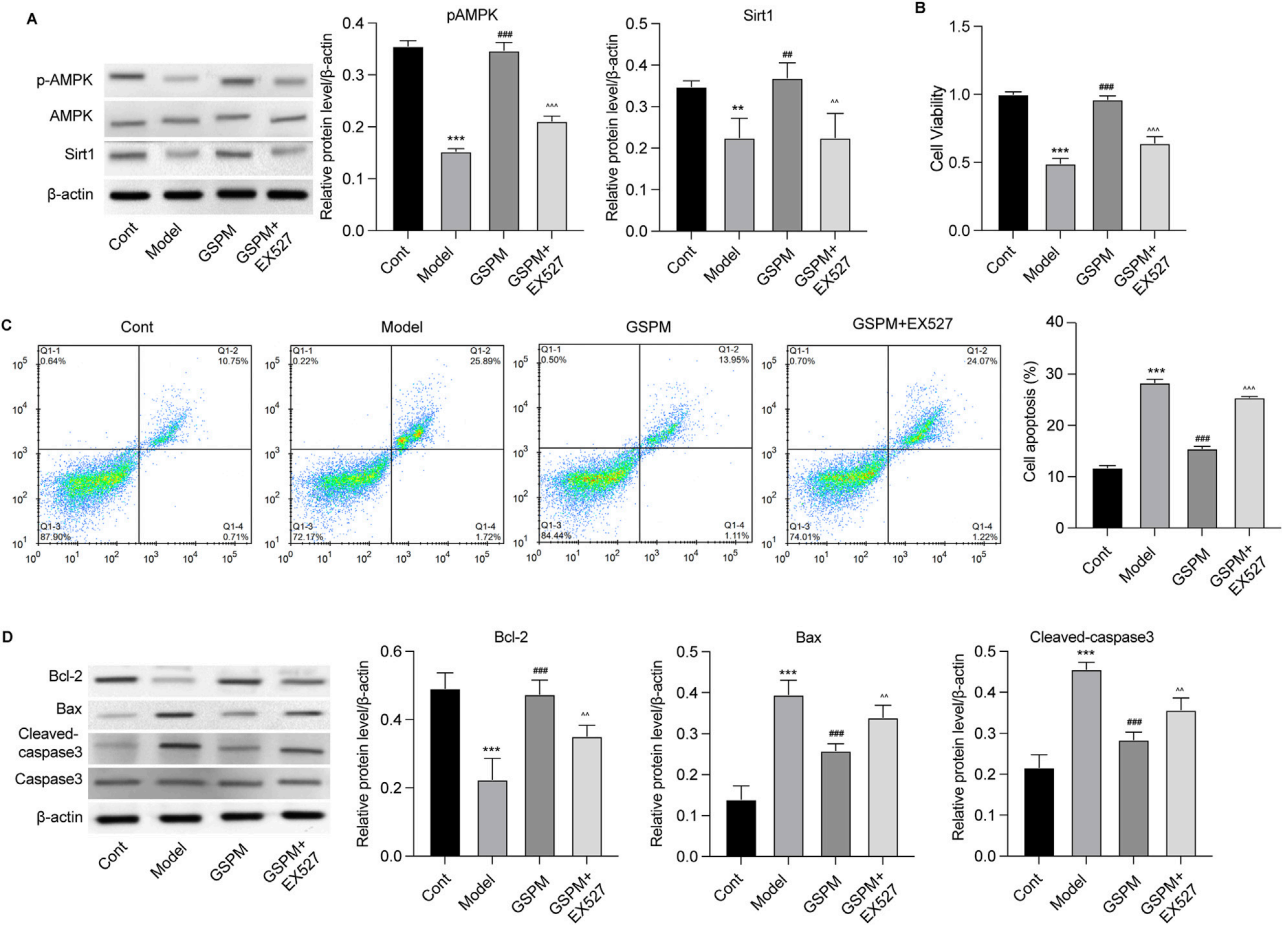
GSPM protects neurons from apoptosis via regulating Sirt1. HT22 cells were cultured with Aβ1-42 to establish an AD model, followed by a 2 h pretreatment with a SIRT1 inhibitor, and subsequent treatment with GSPM. **(A)** Protein levels of phosphorylated AMK, AMPK, and Sirt1 in HT22 cells were detected by Western blotting. **(B)** Cell viability and **(C)** apoptosis were detected by CCK-8 and flow cytometry, respectively. **(D)** Protein levels of Bcl-2, Bax, cleaved-caspase 3, and caspase 3 were measured by Western blotting. *p < 0.05, **p < 0.01, ***p < 0.001 vs. cont group; #p < 0.05, ##p < 0.01, ###p < 0.001 vs. model group; ^p < 0.05, ^^p < 0.01, ^^^p < 0.001 vs. GSPM group.

**FIGURE 8:**
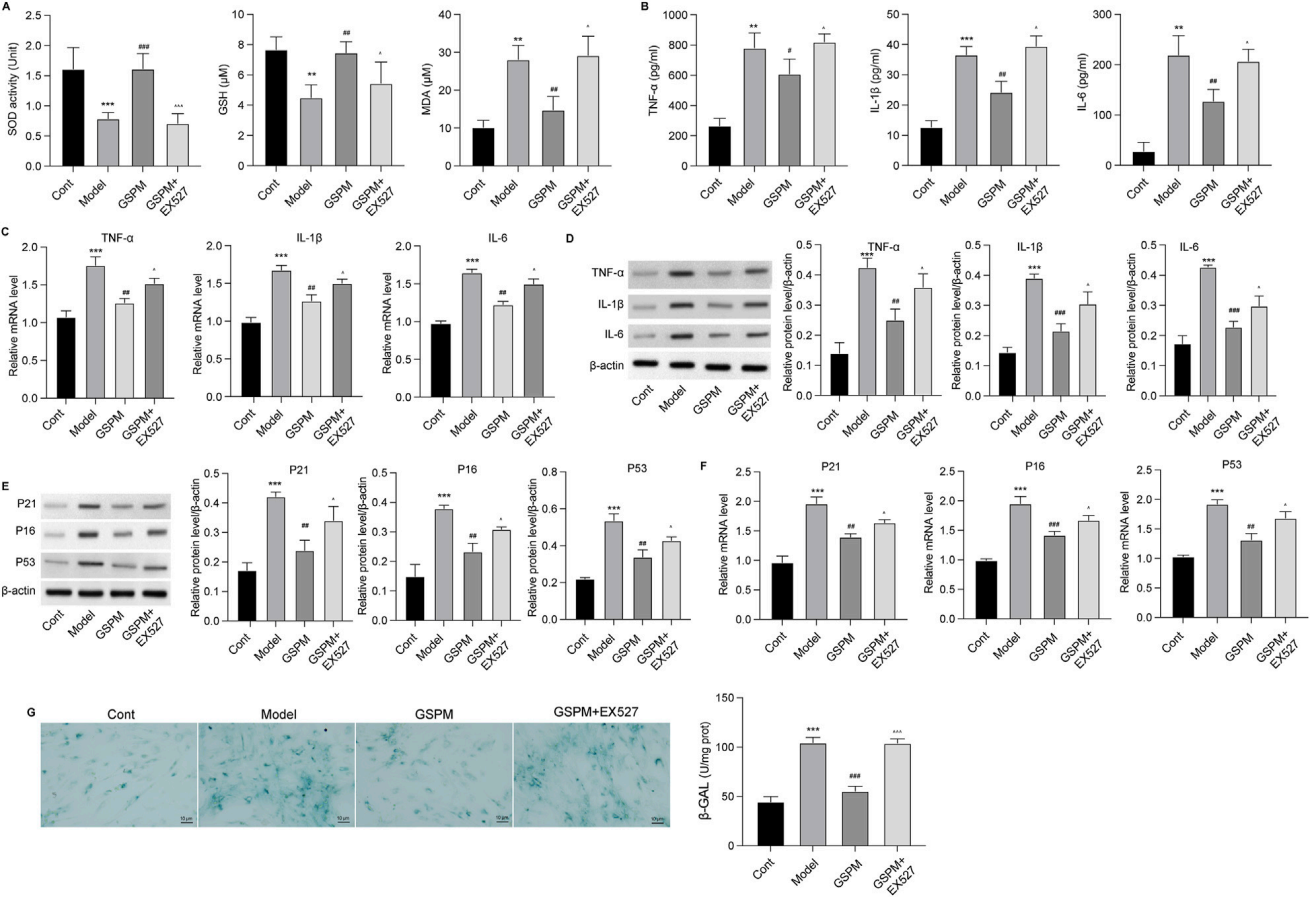
GSPM protects neurons from inflammation and oxidative stress via regulating Sirt1. HT22 cells were cultured with Aβ1-42 to establish an AD model, followed by a 2 h pretreatment with a SIRT1 inhibitor, and subsequent treatment with GSPM. **(A)** The SOD activity, GSH and MDA levels in HT22 neurons were evaluated using commercial assay kits. **(B)** Secretion levels, **(C)** mRNA levels, and **(D)** protein levels of TNF-α, IL-1β, and IL-6 were measured by ELISA, qPCR, and Western blotting, respectively. **(E)** Protein and **(F)** mRNA levels of P21, P16, and P53 were measured by Western blotting and qPCR. **(G)** The SA-β-gal activity of HT22 cells was detected via an SA-β-gal staining assay. *p < 0.05, **p < 0.01, ***p < 0.001 vs. cont group; #p < 0.05, ##p < 0.01, ###p < 0.001 vs. model group; ^p < 0.05, ^^p < 0.01, ^^^p < 0.001 vs. GSPM group.

To confirm the role of Sirt1 as a mediator of GSPM’s neuroprotective effects, we inhibited Sirt1 upon GSPM treatment. As shown in Figure 7A, the Sirt1 inhibitor EX527 significantly reduced AMPK/Sirt1 activation in GSPM-treated HT22 cells and reversed the GSPM-induced increase in cell viability (Figure 7B). Sirt1 inhibition also reversed the GSPM-induced reduction of cell apoptosis (Figure 7C), as well as the increased expression of Bcl-2 and decreased expression of Bax and cleaved-caspase 3 protein in Aβ1-42-stimulated HT22 cells (Figure 7D). Furthermore, EX527 could abolish the GSPM-induced increase of SOD1 and GSH levels, as well as the reduction of MDA level (Figure 8A). In addition, EX527 could reverse the GSPM-mediated suppression of pro-inflammatory factors (TNF-α, IL-1β, and IL-6) (Figures 8B–D) and GSPM-mediated alleviation of neuronal senescence, as demonstrated by increased P21, P16, and P53 expression (Figures 8E, F) and enhanced SA-β-gal staining (Figure 8G).

## Discussion

The potential of TCM in the treatment of AD has been widely recognized in numerous experimental studies and clinical trials. Accumulative studies have indicated that TCM exerts neuroprotective effects through various mechanisms, such as antioxidation, anti-inflammation, and cognitive function improvement, making it a promising strategy for AD treatment (Tan et al., 2022). Among these, extracts from *ginseng* and *P. multiflorum* have garnered particular attention due to their notable neuroprotective properties (Liu et al., 2024). In the present study, we performed HPLC analysis of GSPM and identified 13 active metabolites, of which 7 were derived from *P. multiflorum* (e.g., emodin, polydatin) and 6 from *ginseng* (e.g., ginsenosides Rg1, Re, Rg2). These metabolites have been demonstrated in previous studies to possess anti-inflammatory, antioxidative, and neuroprotective effects, effectively improving the hallmark pathological features of AD. For example, ginsenosides, particularly Rg1, Re, and Rg2, have been widely studied for their ability to prevent memory loss and improve cognitive dysfunction (Liang et al., 2021; Cui et al., 2021). KM et al. demonstrated that ginsenosides Re and Rd could regulate the transporters involved in cholinergic neurotransmission, playing a critical role in neuronal differentiation and injury (Kim et al., 2014). Jing et al. found that ginsenoside Rg2 treatment of Aβ25-35-induced PC12 cells significantly increased cell survival rates and protected against neuronal apoptosis (Cui et al., 2017). Chen et al. reported that tetrahydroxystilbene glucoside and emodin might reduce neuronal apoptosis by regulating histone acetylases (Chen, 2022). Additionally, Chen et al. found that emodin could alleviate nitric oxide-induced damage to neural cells (Chen et al., 2016). These studies provide theoretical support for the potential role of GSPM in AD treatment. This study further investigated the effects of GSPM in an AD mouse model. The results showed that GSPM significantly improved cognitive function, as evidenced by reduced escape latency and increased platform crossings in the Morris water maze. GSPM also alleviated hippocampal neuronal damage and reduced activation of microglia and astrocytes associated with AD plaques, suggesting its neuroprotective effect via neuroinflammation reduction. Additionally, GSPM decreased Aβ accumulation and Tau protein phosphorylation, further improving AD pathology. Moreover, GSPM enhanced antioxidant levels (e.g., SOD, GSH) and suppressed pro-inflammatory cytokine secretion (e.g., TNF-α, IL-1β, IL-6), indicating its role in modulating oxidative stress and inflammation in AD. These findings support GSPM as a promising therapeutic strategy for AD.

Accumulating evidence has suggested that imbalanced metabolism and inflammatory responses may not only originate in the brain but also from gut microbiota (Kau et al., 2011). The gut microbiota actively participates in the functions of the gut-brain axis, influencing cognitive function and related behaviors. This bidirectional communication between the gut microbiome and the brain is referred to as the “microbiota–gut–brain” (MGB) axis (Liu et al., 2022; Chakrabarti et al., 2022). The mechanisms underlying this communication involve neural, immune, endocrine, and metabolic signaling (Wiatrak et al., 2022). Notably, various extracts of botanical drugs have demonstrated neuroprotective effects through modulation of the gut microbiota. For example, *Rheum tanguticum* (Maxim. ex Regel) Balf*.* [Polygonaceae; Rheum tanguticum radix], a commonly used botanical drug in TCM for AD treatment, has been shown to effectively alleviate cognitive impairments and restore gut microbiome homeostasis in AD models (Gao et al., 2022; Zhao et al., 2019). Additionally, Rg1, the primary active metabolite of *ginseng*, exhibits anti-apoptotic, anti-inflammatory, and antioxidant properties, which contribute to cognitive function improvement and microbiota balance restoration in the AD animal model (Guo et al., 2021).

In line with this concept, our study aimed to investigate the effects of GSPM on gut microbiota composition in aging AD mice. Using 16S rRNA sequencing, we observed significant differences in microbial diversity between aging AD and control mice, with high-dose GSPM restoring the microbial composition toward the control group. At the phylum level, high-dose GSPM reduced the increased abundance of *Campilobacterota* and *Firmicutes* observed in the model group. Additionally, GSPM treatment dose-dependently restored the elevated F/B ratio in aging AD mice. These findings suggest that GSPM may promote a healthier gut environment by modulating gut microbiota composition. Dysbiosis, characterized by an imbalance in gut microbiota, is commonly observed in AD and linked to systemic inflammation and gut-brain axis dysfunction (Megur et al., 2020). By restoring microbial balance and reducing pathogenic taxa such as *Campilobacterota* and *Firmicutes*, GSPM may help mitigate dysbiosis in aging AD mice. The F/B ratio is often used as an indicator of gut health, with an elevated ratio associated with gut barrier dysfunction and inflammation. Previous studies have shown that an elevated F/B ratio is linked to weakened epithelial tight junctions, which facilitate the translocation of proinflammatory cytokines to the brain, exacerbating neuroinflammation and cognitive decline (Braniste et al., 2014; Al-Asmakh and Hedin, 2015). In this study, GSPM’s ability to restore the F/B ratio in a dose-dependent manner suggests that it may have therapeutic potential in preventing or mitigating the gut-brain axis dysfunction associated with AD. This restoration of gut health may, in turn, help alleviate neuroinflammation, a key feature of Alzheimer’s disease pathology.

In addition to modulating the F/B ratio, GSPM could increase *Lactobacillus* abundance, which is known for its anti-inflammatory properties. *Lactobacillus* has been shown to produce short-chain fatty acids (SCFAs) that help maintain gut barrier integrity and regulate immune responses (Parada Venegas et al., 2019). The negative correlation of *Lactobacillus* with inflammatory markers, Aβ1-42, p-Tau, and senescence biomarkers suggests that GSPM may reduce neuroinflammation and neuronal damage by promoting the growth of beneficial bacteria. Conversely, the reduced abundance of taxa such as *Oscillibacter*, *Helicobacter*, and *Odoribacter* is noteworthy. Studies have demonstrated that the abundance of *Oscillibacter* was significantly reduced following anti-inflammatory treatment in AD rats with neuroinflammation (Wang J. et al., 2022). Moreover, a reduction of *Oscillibacter* has been evidenced to improve cognitive function and enhance learning and memory abilities, particularly following exercise (Wang et al., 2021). Similarly, *Helicobacter pylori* infection induces systemic immune responses via its outer membrane vesicles, leading to microglial activation and neuroinflammation, thereby increasing the risk of AD (Park and Tsunoda, 2022). These findings suggest that GSPM may alleviate neuroinflammation and improve cognitive function by promoting beneficial gut microbiota while reducing harmful bacterial populations associated with inflammation and oxidative stress.

SIRT1, an NAD + -dependent deacetylase, plays pivotal roles in regulating various biological processes, including aging, apoptosis, and neuroprotection, all of which are critically involved in the pathogenesis of AD (Zhang and Tang, 2023). Activation of SIRT1 has been shown to improve synaptic plasticity, enhance memory, and protect neurons from age-related degeneration (Zhang et al., 2011). Conversely, its suppression contributes to increased neuronal apoptosis and cognitive decline (Park et al., 2021). Consequently, targeting the SIRT1 pathway has emerged as a promising therapeutic strategy for AD. Consistent with these findings, our results further support the critical role of SIRT1 in neuroprotection in AD. Treatment with GSPM significantly upregulated the expression and activation of SIRT1 in Aβ1-42-induced neuronal models. Modulation of the SIRT1 pathway by GSPM resulted in reduced neuronal apoptosis and improved cell viability. Importantly, the neuroprotective effects of GSPM were reversed upon inhibition of SIRT1 using the EX527 inhibitor, thereby confirming that the therapeutic benefits of GSPM are, at least in part, mediated through SIRT1 activation. These findings underscore the potential of GSPM as a therapeutic agent for AD by targeting the SIRT1 signaling pathway. By modulating this pathway, GSPM offers a novel approach to mitigate key pathological features of AD, including neuroinflammation, oxidative stress, and neuronal senescence, which are central to the progression of the disease.

To sum up, our study provides new insights into the neuroprotective mechanisms of GSPM in Alzheimer’s disease (AD), showing its effectiveness in combating AD through the suppression of inflammation, oxidative stress, and neuronal senescence, along with the regulation of the Sirt1 pathway. GSPM also significantly altered the gut microbiota in AD mice, suggesting a potential gut-brain axis modulation. While our findings support GSPM’s therapeutic potential, further research is needed to identify the active extracts and confirm their safety and efficacy in both animal models and clinical trials.

## Data Availability

The original contributions presented in the study are included in the article/Supplementary Material, further inquiries can be directed to the corresponding authors.

## Abbreviations

AD, Alzheimer’s disease; GSPM, *Panax ginseng* C.A.Mey. and *Polygonum multiflorum* Thunb; MAD, malondialdehyde; SOD, superoxide dismutase; GSH, glutathione; T-AOC, Total antioxidant capacity; F/B, *Firmicutes* to *Bacteroidetes*; Sirt1, silent information regulator 1; SAMR1, senescence-accelerated mouse resistant 1; SAMP8, senescence-accelerated mouse prone 8; HE, hematoxylin and eosin; IF, immunofluorescence; TUNEL, TdT-mediated dUTP-biotin nick end-labeling; HPLC, high performance liquid chromatography; TNF-α, tumor necrosis factor-α; IL-1β, interleukin-1β; IL-6, interleukin-6.
