# Supplementary figures and images for: Ginseng and Polygonum multiflorum formula protects brain function in Alzheimer’s disease
Source: Front Pharmacol. 2025 Feb 20;16:1461177. doi: 10.3389/fphar.2025.1461177 (PMC11882532; doi:10.3389/fphar.2025.1461177)

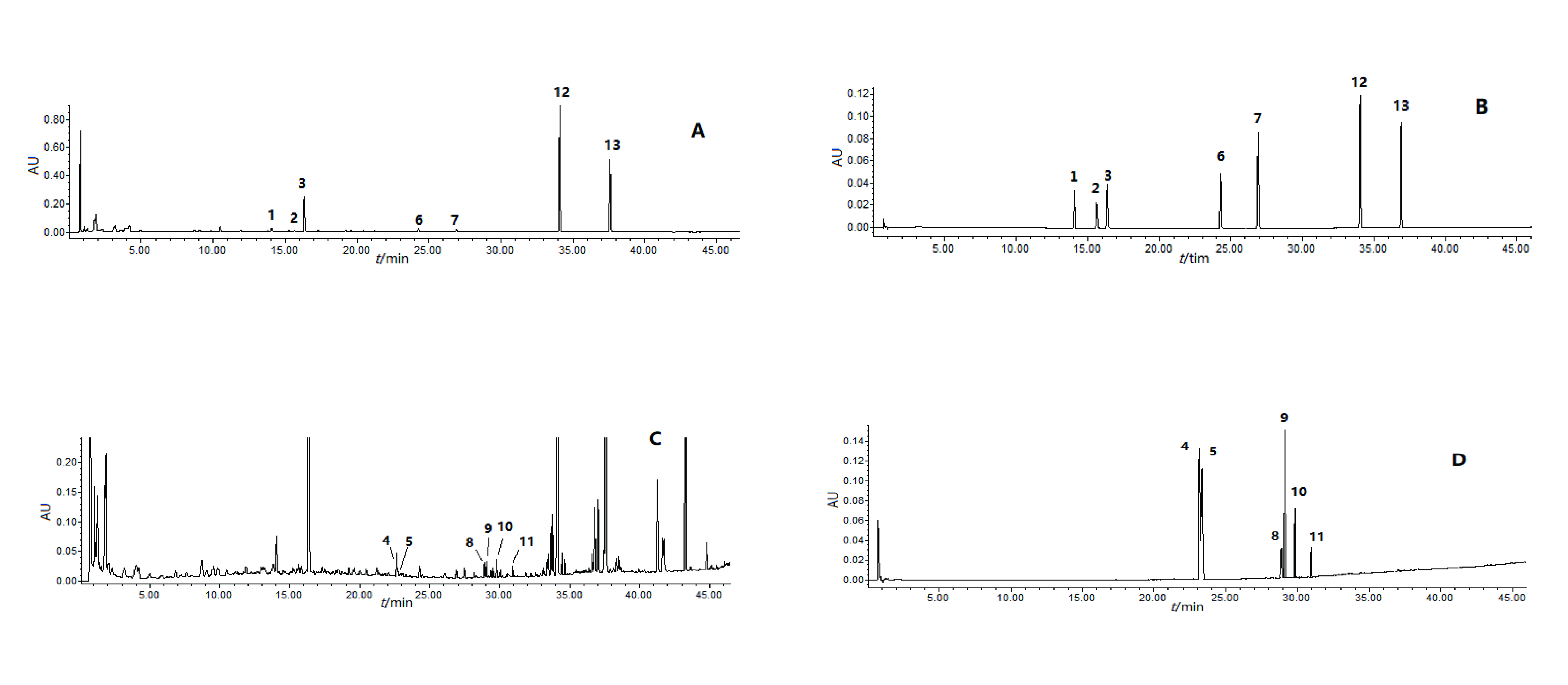

Supplement: Supplementary file 1 [file Image1.tif]
